# Critical review of the impacts of grazing intensity on soil organic carbon storage and other soil quality indicators in extensively managed grasslands

**DOI:** 10.1016/j.agee.2017.10.023

**Published:** 2018-02-01

**Authors:** M. Abdalla, A. Hastings, D.R. Chadwick, D.L. Jones, C.D. Evans, M.B. Jones, R.M. Rees, P. Smith

**Affiliations:** a Institute of Biological and Environmental Sciences, School of Biological Sciences, University of Aberdeen, Aberdeen, AB24 3UU, UK; b School of Environment, Natural Resources and Geography, Bangor University, Bangor, Gwynedd, LL57 2UW, UK; c Department of Botany, School of Natural Sciences, Trinity College Dublin, Dublin 2, Ireland; d Scotland’s Rural College (SRUC) Edinburgh, West Mains Road, Edinburgh, EH93JG, UK

**Keywords:** Grazing, Soil organic carbon, Grassland, Grazing intensity, Total nitrogen

## Abstract

Livestock grazing intensity (GI) is thought to have a major impact on soil organic carbon (SOC) storage and soil quality indicators in grassland agroecosystems. To critically investigate this, we conducted a global review and meta-analysis of 83 studies of extensive grazing, covering 164 sites across different countries and climatic zones. Unlike previous published reviews we normalized the SOC and total nitrogen (TN) data to a 30 cm depth to be compatible with IPCC guidelines. We also calculated a normalized GI and divided the data into four main groups depending on the regional climate (dry warm, DW; dry cool, DC; moist warm, MW; moist cool, MC). Our results show that taken across all climatic zones and GIs, grazing (below the carrying capacity of the systems) results in a decrease in SOC storage, although its impact on SOC is climate-dependent. When assessed for different regional climates, all GI levels increased SOC stocks under the MW climate (+7.6%) whilst there were reductions under the MC climate (−19%). Under the DW and DC climates, only the low (+5.8%) and low to medium (+16.1%) grazing intensities, respectively, were associated with increased SOC stocks. High GI significantly increased SOC for C4-dominated grassland compared to C3-dominated grassland and C3-C4 mixed grasslands. It was also associated with significant increases in TN and bulk density but had no effect on soil pH. To protect grassland soils from degradation, we recommend that GI and management practices should be optimized according to climate region and grassland type (C3, C4 or C3-C4 mixed).

## Highlights

• The impact of grazing on SOC is climate-dependent.
• Grazing increases SOC for C4 but decreases it for C3 and C3-C4 mixed grasslands.
• Grazing increases TN and BD but has no effect on soil pH.

## 1 Introduction

Grasslands cover approximately 40% of the earth's land surface (Wang and Fang, 2009) and represent about 70% of the agricultural area (Conant, 2012). They contain about 10% of terrestrial biomass and make a contribution of about 20–30% to the global pool of soil organic carbon (SOC) (Scurlock and Hall, 1998, Conant et al., 2001). Grasslands have some potential to sequester atmospheric CO_2_ as stable carbon (C) in the soil (Reid et al., 2004) and hence could contribute to mitigation of climate change (Allard et al., 2007). However, the accumulation and storage of C in grasslands is influenced by many factors, especially biotic factors e.g. grazing intensity (GI), animal type and grass species (Conant et al., 2001, Olff et al., 2002, Jones and Donnelly, 2004, McSherry and Ritchie, 2013). Nevertheless, although grasslands have high SOC contents, recent studies have suggested that intensive livestock management has led to C losses from many grasslands around the world and thereby, grassland soils could become a source rather than a sink for greenhouse gas (GHG) emissions (Janzen, 2006, Ciais et al., 2010, Powlson et al., 2011). Grazing intensity has the potential to modify soil structure, function and capacity to store organic carbon (OC) (Cui et al., 2005) and could significantly change grassland C stocks (Cui et al., 2005). As SOC has a major influence on soil physical structure and a range of ecosystem services (e.g. nutrient retention, water storage, pollutant attenuation), its reduction could lead to reduced soil fertility and consequently, land degradation (Rounsevell et al., 1999). These effects may also be magnified if SOC loss rates are magnified by climate change (Lal, 2009). However, investigating the effects of GI on SOC is hampered by the heterogeneity in grassland types and variations in environmental factors among sites. This is exacerbated by the fact that all previous published meta-analyses studies on this topic (e.g. McSherry and Ritchie, 2013, Lu et al., 2017, Zhou et al., 2017) pooled the data of different studies together without considering the differences in soil depth at which the SOC and TN were measured, thus producing highly uncertain/contradictory results.

High GI could indirectly alter grass species composition (Cingolani et al., 2005) by decreasing water availability (Pineiro et al., 2010). This decreases plant community composition, aboveground biomass, leaf area and light interception and thereby, net primary production (NPP) (Manley et al., 1995; Pineiro et al., 2010). However, according to Derner and Schuman (2007), Pineiro et al. (2010) and McSherry and Ritchie (2013), high GI can increase soil C sequestration but only when mean annual precipitation is 600 mm or less, and with different responses observed in different soil types. Grazing intensity has also been shown to increase root C contents (a primary control of SOC formation) at the driest and wettest sites, but decrease root C contents at intermediate precipitation levels (400 mm–850 mm) (Pineiro et al., 2010). Wang et al. (2017) reported that the compositions of plant species and soil condition in the Tibetan pastures were not only affected by GI but also by the local environmental factors. Moreover, Russell et al. (2013) suggest that grazing at high intensity for a short period of time was effective at increasing soil organic matter and diversity in forage species composition. On the other hand, overgrazing to the point of stripping surface vegetation can result in soil-degradation and loss of the fertile topsoil, especially where precipitation is low and evaporation is high (Xie and Wittig, 2004).

Furthermore, high GI can alter SOC by changing the competitive abilities of different microbial phyla because of the link between GI, SOC availability and ecosystem functions (Eldridge et al., 2017). However, Eldridge and Delgado-Baquerizo (2017) suggest that, the relationship between GI and SOC is generally non-linear. Previous studies have found mixed results (Derner et al., 2006, McSherry and Ritchie, 2013, Zhou et al., 2017), with some showing increases (Reeder and Schuman, 2002, Li et al., 2011, Silveira et al., 2014), while others show no effect (Frank et al., 2002, Shrestha and Stahl, 2008, Cao et al., 2013) or decreases (Zuo et al., 2008, Golluscio et al., 2009, Reszkowska et al., 2011, Qiu et al., 2013) in SOC stocks. The review by McSherry and Ritchie (2013) showed that GI effects on SOC are highly context-specific where higher GI increased SOC on C4-dominated and C4-C3 mixed grasslands, but decreased SOC in C3-dominated grasslands. Other recent reviews by Lu et al. (2017) and Zhou et al. (2017) found that high GI significantly decreased belowground C and N pools. They found that GI interacts with elevation and mean annual temperature (Lu et al., 2017) or with soil depth, livestock type and climatic conditions (Zhou et al., 2017).

Understanding the impacts of GI on SOC accumulation and storage in grasslands is crucial to provide the most effective soil C management options. However, although all of these previous reviews are valuable, scientific understanding would be improved by normalizing the sampling depth and GI. In this study, to be compatible with the IPCC guidelines, reduce these errors and make a comprehensive evaluation for GI we have normalized the soil depth for all studies to 30 cm using a quadratic density function based on Smith et al. (2000) and calculated a normalized GI. The major objective of this meta-analysis was to investigate the impacts of GI on SOC in extensively grazed grassland soils at a global scale. Additionally, and because of its importance for C biogeochemistry, we considered the impacts of GI on total nitrogen (TN) and other soil properties (mainly pH and bulk density) in grasslands. We also investigated whether spatial variations in climate determine the ecological effects of grazing practices on SOC in grasslands. The specific hypotheses we critically evaluated are as follows: 1) higher GI decreases SOC and TN in soils; 2) the impacts of GI on SOC are modified by environmental and biotic factors; and 3) the effects of GI on SOC stocks depends on climatic zone and soil texture.

## 2 Materials and methods

### 2.1 Data collection

**Fig. 1:**
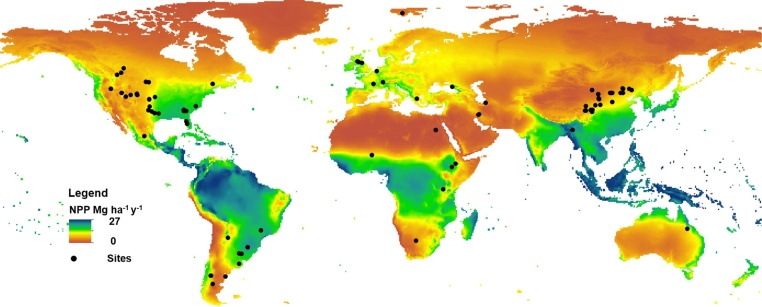
Map of mean Net Primary Production (NPP) in mg C ha^−1^ y^−1^ derived from the mean annual temperature and mean annual precipitation using the Miami model with the locations of experimental sites considered in this paper.

To collect published studies that have investigated the impacts of GI on SOC and other selected soil properties (TN, pH and BD) under grassland, we performed a comprehensive search on the Web of Science database (accessed between January 2015 and July 2017) using the following keywords: grazing; soil organic carbon; grassland; GI; total nitrogen and carbon sequestration. In an attempt to have the best possible coverage; we also checked all references in the papers found in the Web of Science search. Only studies which were longer than one year and measured SOC or TN were selected. We also accounted for the differences in grass growing seasons at each experimental site. Our searches resulted in 83 studies that investigated the impacts of grazing on SOC and other selected soil properties; carried out at 164 sites covering different countries; climatic zones and management systems (Fig. 1). The studies were segregated into four groups depending on the regional climatic zones (dry cool (DC); dry warm (DW); moist cool (MC) and moist warm (MW)).

We defined the climatic zones based on thermal and moisture regimes: cool, warm, dry, and moist zone according to Smith et al. (2008). The cool zone covers the temperate (oceanic, sub-continental, and continental) and boreal (oceanic, sub-continental and continental) areas, whilst the warm zone covers the tropics (lowland and highland) and subtropics (summer rainfall, winter rainfall, and low rainfall) areas. The dry zone includes the areas where the annual precipitation is equal or below 500 mm, whilst the moist zone includes areas where the annual precipitation is above 500 mm. Coordinates, grass type (i.e. shrubby, woody, steppe, and prairie), annual mean climatic conditions as well as grazing details, soil texture, original depth (OD), initial and final BD and pH, changes in SOC and TN (kg m^−2^); values were added where available or were designated plus (+) for increased and minus (−) for decreased, as shown in Table 1, Table 2, Table 3, Table 4.

**Table 1:** Published studies on the impacts of grazing on SOC and other soil properties in the moist/cool climatic zone.

Coordinates (country/state)	Grass type	C3/C4/M	Grazing intensity	Type of animal	Duration (year)	Soil texture	iBD (g cm^−3^)	ipH^a^	MAAT (°C)	MAP (mm)	OD (cm)	ΔSOC kg m^−2 ^C (0–30 cm)	ΔTN kg m^−2^ N (0–30 cm)	fBD (g cm^−3^)	fpH	Added N	Ref
99°47′N, 33°37′E (CN)	Alpine meadow	C3	HG	Yaks	3.0	ND	1.6	6.8	−1.3	590	0–20	−0.8	−	1.9	6.7	ND	1
	Alpine meadow	C3	MG	Yaks	3.0	ND	1.6	6.8	−1.3	590	0–20	−1.0	−	1.8	6.8	ND	1
	Alpine meadow	C3	LG	Yaks	3.0	ND	1.6	6.8	−1.3	590	0–20	−1.4	−	1.7	6.9	ND	1
	Alpine meadow	C3	MG^+^	Yaks	3.0	ND	1.6	6.8	−1.3	590	0–20	−1.2	−	2.2	7.0	ND	1
33°03′N, 102°36′E (CN)	Alpine meadow	C3	HG	Yaks	9.0	Loamy sand	ND	ND	1.1	752	0–30	+	+	ND	ND	0	2
	Alpine meadow	C3	MG	Yaks	9.0	Loamy sand	ND	ND	1.1	752	0–30	+	+	ND	ND	0	2
	Alpine meadow	C3	LG	Yaks	9.0	Loamy sand	ND	ND	1.1	752	0–30	+	+	ND	ND	0	2
46°37′N, 07°15′E (CH)	Subalpine Pasture	C3	HG	Cows	150.0	Loamy sand	0.9	4.9	6.0	1250	0–25	−0.2	−0.1	0.9	4.8	ND	3
	Bare	–	HG	Cows	150.0	Loamy sand	0.9	4.9	6.0	1250	0–25	−1.6	−0.1	1.1	5.1	ND	3
45°43′N, 03°01′E (FR)	Semi-natural monolith	C3	HG	Sheep	14.0	Sandy soil	ND	5.6	ND	637	0–20	−	ND	ND	ND	U	4
45°43′N, 03°01′E (FR)	Semi-natural monolith	C3	HG	Sheep	14.0	Sandy soil	ND	5.6	ND	637	0–20	−	ND	ND	ND	U	5
33°42′N, 102°07′E (CN)	Alpine meadow	C3	HG	Sheep/yaks	10.0	ND	0.9	ND	12.0	620	0–15	+	+	1.0	ND	0	6
	Alpine meadow	C3	MG	Sheep/yaks	10.0	ND	0.9	ND	12.0	620	0–15	+	+	0.9	ND	0	6
	Alpine meadow	C3	LG	Sheep/yaks	10.0	ND	0.9	ND	12.0	620	0–15	+	+	0.9	ND	0	6
	Alpine meadow	C3	HG	Sheep/yaks	10.0	ND	0.9	ND	12.0	620	0–15	+	+	1.0	ND	0	6
33°56′N, 102°52′E (CN)	Wet meadow	C3	HG^*^	Yaks/sheep	5.0	ND	0.4	8.0	0.9	657	0–10	−7.1	0.0	0.4	8.0	0	7
33°55′N, 102°49′E (CN)	Meadow	C3	HG^*^	Yaks/sheep	5.0	ND	0.5	7.6	0.9	657	0–10	1.5	−0.3	0.6	7.8	0	7
33°55′N, 102°52′E (CN)	Marsh	C3	HG^*^	Yaks/sheep	5.0	ND	0.3	8.0	0.9	657	0–10	−1.7	0.0	0.3	7.8	0	7
32°49′N, 102°00′E (CN)	Alpine meadow	C3	HG	Yaks	5.0	Silt loam	0.8	5.5	1.4	648	0–10	−0.3	−0.1	1.1	5.6	0	8
55°49′N, 03°49′W (UK)	Ryegrass/White clover	C3	HG	Ewes/lambs/goats/cows	16.0	Loamy sand	0.9	6.0	ND	1265	0–15	+	−	ND	ND	90	9
	Ryegrass/White clover	C3	HG	Ewes/lambs/goats/cows	16.0	Loamy sand	0.9	5.5	ND	1057	0–15	−	+	ND	ND	90	9
54°18′N, 02°36′E (UK)	Acidic grassland	C3	HG	Ewes/cows	7.0	Sandy soil	0.0	4.5	ND	1840	0–20	+	0.0	0.0	ND	0	10
39°169′N, 22°71′E (EL)	Grassland	C4	HG	Livestock	ND	Sandy/sandy Clay/sandy clay loam	ND	ND	ND	ND	0–20	−	ND	ND	ND	ND	11
	Grassland	C4	MG	Livestock			ND	ND	ND	ND	0–20	+	ND	ND	ND	ND	11
56°16^′^N, 04°24′W (UK)	Fine grained mosaic	C3	HG	Sheep	100.0^s^	Organic soil	ND	ND	ND	1344	0–15	−	ND	ND	ND	ND	12
	Fine grained mosaic	C3	LG	Sheep	100.0^s^	Organic soil	ND	ND	ND	1344	0–15	+	ND	ND	ND	ND	12
33°59^′^N, 102°34′E (CN)	Alpine meadow	C3	HG	Yaks/sheep	3.0	Sandy soil	ND	ND	ND	620	0–15	−	ND	ND	ND	ND	13

MAAT – mean annual air temperature (°C) and MAP – mean annual precipitation. Changes in SOC (ΔSOC) and total nitrogen (ΔTN) were calculated at 0–30 cm depth using the original depth in each paper and converted into kg m^−2^ of C or N, respectively. ^a^ = Different methods were used to measure soil pH using pH probe/meter in deionized water or 0.01 M CaCl_2_ in 1:1 and 1:2, or 1:5 (v: v) soils: solution ratios. Added N fertilizer is in kg N ha^−1^. OD = original measurements depth. BD = initial bulk density; fBD = bulk density after grazing; ipH = initial pH; fpH = pH after grazing; ^S^ = simulation study; HG = high grazing; MG = medium grazing; LG = low grazing; * = originally described as free grazing; MG^+^ = originally described as native grazing; ND = no data; negative sign = decreased; positive sign = increased; N = added nitrogen fertilizer in kg N ha^−1^; U = urine (5 g N m^−2^). SOC = soil organic carbon; ΔSOC = difference in soil organic carbon between un-grazed and grazed site; ΔTN = difference in total nitrogen between un-grazed and grazed site. C3 = C3 crop; C4 = C4 crop and M = mixed C3/C4 crops. CH = Switzerland; CN = China; EL = Greece; FR = France; UK = United Kingdom. Ref = reference: 1 = Dong et al. (2012); 2 = Gao et al. (2007); 3 = Hiltbrunner et al. (2012); 4 = Klumpp et al. (2007); 5 = Klumpp et al. (2009); 6 = Li et al. (2011); 7 = Luan et al. (2014); 8 = Ma et al. (2016); 9 = Marriott et al. (2010); 10 = Medina-Roldan et al. (2012); 11 = Pappas and Koukoura (2011); 12 = Smith et al. (2014); 13 =.

**Table 2:** Published studies on the impacts of grazing on SOC in the moist/warm climatic zone.

Coordinates (country/state)	Grass type	C3/C4/M	Grazing intensity	Type of animal	Duration (year)	Soil texture	iBD^a^ (g cm^−3^)	ipH	MAAT (°C)	MAP (mm)	OD (cm)	ΔSOC kg m^−2^ C (0–30 cm)	ΔTN kg m^−2^ N (0–30 cm)	fBD (g cm^−3^)	fpH	Added N	Ref
24°43′S, 63°17′W (AR)	Subtropical woodland/grasses	M	HG	Cattle/goats	ND	Sandy/loam	0.9	7.0	ND	550	0–20	−	−	0.9	6.97	ND	1
	Subtropical woodland/grasses	M	HG	Cattle/goats	ND	Coarse silt/loam	0.9	7.0	ND	550	0–20	−	−	1.1	6.94	ND	1
	Subtropical woodland/grasses	M	HG	Cattle/goats	ND	Silty clay/loam	0.9	7.0	ND	550	0–20	−	−	1.2	6.95	ND	1
31°54′S, 58°15′W (UY)	Mesic grassland	C3	MG	Cows	25.0	Clay soil	1.3	ND	17.4	1099	0–30	−	ND	1.4	ND	ND	2
	Mesic grassland	C3	MG	Cows	25.0	Clay soil	1.3	ND	17.4	1099	30–100	−	ND	1.4	ND	ND	2
36°30′S, 58°30′W (AR)	Grasses & sedges	C3	LG	Cows	14.0	Loamy soil	1.2	ND	15.0	1007	0–10	+	+	1.2	ND	ND	3
28°56′S, 54°20′W (BR)	Black oat/Italian ryegrass	C3	HG	Cows	10.0	Clay soil	1.2	4.2	19.0	1850	0–20	−	ND	ND	ND	ND	4
	Black oat/Italian ryegrass	C3	MG	Cows	10.0	Clay soil	1.2	4.2	19.0	1850	0–20	+	ND	ND	ND	ND	4
	Italian Ryegrass/Black oat	C3	LG	Cows	10.0	Clay soil	1.2	4.2	19.0	1850	0–20	+	ND	ND	ND	ND	4
39°05′N, 96°35′W (USA)	Tall grass	C4	MG	Cows	36.0	Silty clay loam	1.1	6.3	12.5	835	0–30	−0.7	ND	1.1	ND	ND	5
38°52′N, 99°23′W (USA)	Mid grass	C3	MG	Cows	36.0	Silt loam	0.9	8.3	11.9	588	0–30	−0.7	ND	1.0	ND	ND	5
24°43′N, 93°50′E (IN)	Subtropical grass	C4	HG	Cows	1.0	Clayey loam	1.2	5.9	12.9	1522	0–10	−	−	1.2	6.0	ND	6
	Subtropical grass	C4	MG	Cows	1.0	Clayey loam	1.2	5.9	12.9	1522	0–10	+	+	1.2	5.6	ND	6
41°02′S, 71°04′W (AR)	Wet meadow	C3	HG	Sheep	2.0	Organic soil	4.3	7.9	8.3	650	0–100	−	ND	1.0	8.3	ND	7
41°02′S, 71°04′W (AR)	Mesic meadow	C3	HG	Sheep	2.0	Sandy loam	4.3	7.9	8.3	650	0–100	−	ND	1.2	8.0	ND	7
46°46′N, 100°50′W (USA)	Mixed prairie	M	HG	Steers	76.0	Silt Loam	ND	ND	ND	ND	0–30	0.2	ND	ND	ND	ND	8
	Mixed prairie	M	HG	Steers	76.0	Silt Loam	ND	ND	ND	ND	0–107	−0.2	−0.1	ND	ND	ND	8
	Mixed prairie	M	MG	Steers	76.0	Silt Loam	ND	ND	ND	ND	0–30	−0.8	ND	ND	ND	ND	8
	Mixed prairie	M	MG	Steers	76.0	Silt Loam	ND	ND	ND	ND	0–107	−5.4	−0.1	ND	ND	ND	8
33°52′N, 83°25′W (USA)	Tall fescue pasture	C4	HG	Angus cattle	7.0	Sandy loam/loam/sandy clay loam	ND	6.5	16.5	1250	0–20	+	ND	ND	ND	93	9
33°22′N, 83°24′W (USA)	Bermuda grass	C3	HG	Angus steers	7.0	Sandy loam	ND	6.5	16.5	1250	0–90	+	0.1	ND	ND	200	10
			LG	Angus steers	5.0	Sandy loam	ND	6.5	16.5	1250	0–90	+	0.1	ND	ND	200	10
33°22′N, 83°24′W (USA)	Bermuda grass	C3	HG	Angus steers	5.0	Sandy loam	ND	6.5	16.5	1250	0–90	1.2	+	ND	ND	470	11
	Bermuda grass	C3	LG	Angus steers	5.0	Sandy loam	ND	6.5	16.5	1250	0–90	2.4	+	ND	ND	470	11
33°52′N, 83°25′W (USA)	Tall fescue bermudagrass	C4	HG	Angus cattle	14.0	Sandy loam/loam/sandy clay loam	1.5	6.5	16.5	1250	0–200	3.0	−0.3	1.5	ND	93	12
33°52′N, 83°25′W (USA)	Tall fescue bermudagrass	C4	HG	Angus cattle	12.0	Sandy loam/loam/sandy clay loam	1.2	7.0	16.5	1250	0–2.5	+	ND	ND	ND	93	13
	Tall fescue bermudagrass	C4	HG	Angus cattle		Sandy loam/ loam/sandy clay loam	1.2	7.0	16.5	1250	2.5–7.5	+	ND	ND	ND	93	13
	Tall fescue bermudagrass	C4	HG	Angus cattle		Sandy loam/loam/sandy clay loam	1.2	7.0	16.5	1250	7.5–15	+	ND	ND	ND	93	13
	Tall fescue bermudagrass	C4	HG	Angus cattle		Sandy loam/loam/sandy clay loam	1.2	7.0	16.5	1250	5–30	+	ND	ND	ND	93	13
35°25^′^N, 99°05′W (USA)	Grass prairie	C4	HG	Livestock	100.0	Silty clay loam	ND	7.8	ND	766	0–10	−	−	ND	7.8	ND	14
	Grass prairie	C4	MG	Livestock	100.0	Silty clay loam	ND	7.8	ND	766	0–10	−	−	ND	7.6	ND	14
21°18′S, 48°18′W (BR)	Brachiaria grass	C3	HG^c^	Cows	1.0	Clayey soil	ND	4.9	21	1230	0–5	+	−	ND	5.0	150	15
	Brachiaria grass	C3	MG^R^	Cows	1.0	Clayey soil	ND	4.9	21	1230	0–5	+	+	ND	5.2	150	15
99°51′E, 35°32′N (CN)	Winter pasture	C3	MG	Yaks	7.0	ND	ND	ND	ND	582	0–5	1.8	ND	ND	ND	ND	16
	Winter pasture	C3	MG	Yaks	7.0	ND	ND	ND	ND	582	5–15	+	ND	ND	ND	ND	16
13°15′N, 02°18′E (NE)	Rangeland	C3	HG	Sheep/goats	4.0	Sandy soil	1.6	4.9	ND	575	0–30	−	−	1.6	5.3	ND	17
		C3	MG	Sheep/goats	4.0	Sandy soil	1.6	4.9	ND	575		−	−	1.6	5.5	ND	17
32°00′S, 57°08′W &	Grasslands (HL)	C3	HG	Cows	100.0	MS	ND	6.0	17.3	1406	0–100	−1.6	−	ND	ND	ND	18
31°50′S, 58°17′W &	Grasslands	C3							18.9	1300	0–100	−1.6	−	ND	ND	ND	18
33°52′S, 55°33′W &	Grasslands	C3							16.3	1161	0–100	−1.6	−	ND	ND	ND	18
33°19′S, 56°58′W &	Grasslands	C3							17.4	1099	0–100	−1.6	−	ND	ND	ND	18
36°30′S, 58°30′W(AR; UY)	Grasslands	C3							14.9	861	0–100	−1.6	−	ND	ND	ND	18
	Grasslands (LL)	C3										1.8	−	ND	ND	ND	18
	Grasslands (SL)	C3										1.8	+	ND	ND	ND	18
20°34′S, 146°07′E (AU)	Tropical grasses/shrubs	C4	HG	Steers	12.0^S^	Clayey soil	ND	7.0	ND	617	0–30	+	ND	ND	ND	ND	19
		C3	LG	Steers	12.0^S^	Clayey soil	ND	7.0	ND	617	0–30	+	ND	ND	ND	ND	19
31°50^′^N, 51°14′E (IR)	Rangeland	C3	HG	Sheep/goats	0.5	Silty clay	1.5	6.9	10.7	225	0–15	−	−	1.5	7. 5	ND	20
34°50′E, 02°25′S (TZ)	Acacia tortilis/grass	C4	HG	Gazelles/buffalo/zebra	5.0	Silty/clay/sandy	1.0	ND	ND	650	0–10	−	ND	ND	ND	ND	21
		C4	MG	Gazelles/buffalo/zebra	5.0	Silty/clay/sandy	1.0	ND	ND	650	0–10	+	ND	ND	ND	ND	21
		C4	LG	Gazelles/buffalo/zebra	5.0	Silty/clay/sandy	1.0	ND	ND	650	0–10	+	ND	ND	ND	ND	21
28°60′–28°63′N & 82°36′–82°38′W (USA)	Tropical grass	C4	HG	Cows/Calves	2.0	Fine sand	1.5	6.3	ND	1471	0–40	−0.1	ND	1. 5	6.3	ND	22
27°35′N, 81°55′W (USA)	Improved pasture	C3	MG	Cattle	1.0	Sandy soil	ND	5.5	1650	1650	0–20	+	ND	ND	ND	ND	23
	Silvopasture	C4	MG	Cattle	1.0	Sandy soil	ND	5.5		1650	0–20	+	ND	ND	ND	ND	23
	Rangeland	C4	MG	Cattle	1.0	Sandy soil	ND	5.5		1650	0–20	+	ND	ND	ND	ND	23
98°08’N, 33°16’W (USA)	Tall grass Prairie	C4	HG	Cows	2.0	Clay loam	0.9	7.9	18.1	820	0–90	−	1.4	1.1	7.6	0	24
	Tall grass	C4	MG^P^	Cows	2.0	Clay loam	0.9	7.9	18.1	820	0–90	−	1.4	0.9	7.8	0	24
	Prairie		LG	Cows	2.0	Clay loam	0.9	7.9	18.1	820	0–90	−	1.5	1.0	7.7	0	24
09°20’N, 40°20’E (ET)	Open grass	C4	HG^x^	Abernosa Cattle	40.0	Sandy soil	ND	6.74.0	26.0	512	0–10	−	−	ND	6.4	ND	25
07°47’N, 38°40’E (ET)	Open grass	C4	HG^x^	Borana cattle	40.0	Sandy soil	ND	8.2	21.0	734	0–10	−	−	ND	8.0	ND	25
ND (USA)	Bermuda grass	C4	HG	Cows/Calves	32.0	Fine sandy loam	ND	6.2	19.0	1160	0–15	+	+	ND	ND	224–350	26
	Bermuda grass	C4	LG	Cows/Calves		Fine sandy loam	ND	6.2	19.0	1160	0–15	+	+	ND	ND		26
35°38’N, 78°05’W (USA)	Ryegrass/sorghum	C3	HG^x^	Cows	40.0	Loamy sand	1.2	5.3	21.0	1220	0–10	+	+	1.2	5.5	ND	27

MAAT – mean annual air temperature (°C) and MAP – mean annual precipitation. Changes in SOC (ΔSOC) and total nitrogen (ΔTN) were calculated at 0–30 cm depth using the original depth in each paper and converted to kg m^−2^ of C or N, respectively. ^a^ = Different methods were used to measure soil pH using pH probe/meter in deionized water or 0.01 M CaCl_2_ in 1:1 and 1:2, or 1:5 (v: v) soils: solution ratios. Added N fertilizer is in kg N ha^−1^. OD = original measurements depth. iBD = initial bulk density; fBD = bulk density after grazing; ipH = initial pH; fpH = pH after grazing; HG = = high grazing; MG = medium grazing; LG = low grazing; NG = native grazing i.e. 2.50 heads ha^−1^ estimated by comparison with control; * = originally described as free grazing; ^R^ = originally described as rotational grazing; ^c^ = originally described as continuous grazing; ^P^ = originally described as multi-paddock grazing; SG = Series of grazing (e.g. LG, MG, HG). ^S^ = Simulation study; ND = no data; SOC = soil organic carbon. Sp. = species; negative sign = decreased; positive sign = increased; N = added nitrogen fertilizer. ΔSOC = difference in soil organic carbon between un-grazed and grazed site; ΔTN = difference in total nitrogen between un-grazed and grazed site. HL = high land; LL = low land; SL = shallow land. ^x^ = low grazing was considered as control. C3 = C3 crop; C4 = C4 crop and M = mixed C3/C4 crops AR = Argentina; AU = Australia; BR = Brazil; CN = China; ET = Ethiopia; IN = India; IR = Iran; NZ = New Zealand; NE = Niger; TZ = Tanzania; USA = United States of America; UY = Uruguay. Ref. = reference: 1 = Abril and Bucher (1999); 2 = Altesor et al. (2006); 3 = Chaneton & Lavado (1996); 4 = Da Silva et al. (2014); 5 = Derner et al. (2006); 6 = Devi et al. (2014); 7 = Enriquez et al. (2015); 8 = Frank et al. (1995); 9 = Franzluebbers and Stuedemann (2002); 10 = Franzluebbers and Stuedemann (2005); 11 = Franzluebbers and Stuedemann (2009); 12 = Franzluebbers et al. (2000a); 13 = Franzluebbers et al. (2000b); 14 = Fuhlendorf et al. (2002); 15 = Garcia et al. (2011); 16 = Hafner et al. (2012); 17 = Hiernaux et al. (1999); 18 = Pineiro et al. (2009); 19 = Pringle et al. (2011); 20 = Raiesi and Riahi (2014); 21 = Ritchie (2014); 22 = Sigua et al. (2009); 23 = Silveira et al. (2014); 24 = Teague et al. (2011); 25 = Tessema et al. (2011); 26 = Wright et al. (2004); 27 = Yi et al. (2014).

**Table 3:** Published studies on the impacts of grazing on SOC in the dry/cool climatic zone.

Coordinates (country/state)	Grass type	C3/C4/M	Grazing intensity	Type of animal	Duration (year)	Soil texture	iBD (g cm^−3^)	ipH^a^	MAAT (°C)	MAP (mm)	OD (cm)	∆SOC kg m^−2^ C (0–30 cm)	∆TN kg m^−2^ N (0–30 cm)	fBD (g cm^−3^)	fpH	Added N	Ref
43°38′N, 116°42′E (CN)	Steppe grass	C4	SG	Sheep	9.0	Coarse soil	ND	ND	0.0	398	0–20	−	−	ND	ND	0	1
37°36′N, 111°53′E (CN)	Desert steppe	C4	HG	Sheep	4.0	Loam/sandy loam	ND	ND	3.4	280	0–45	−0.4	ND	ND	ND	0	2
	Desert steppe	C4	MG	Sheep	4.0	Loam/sandy loam	ND	ND	3.4	280	0–45	−0.1	ND	ND	ND	0	2
	Desert steppe	C4	LG	Sheep	4.0	Loam/sandy loam	ND	ND	3.4	280	0–45	−0.1	ND	ND	ND	0	2
43°32′N, 116°40′E (CN)	Semiarid steppe	C4	LG	Sheep	20.0	Sandy loam	1.2	7.4	0.2	350	0–60	0.2	+	1.2	7.2	0	3
	Semiarid steppe	C4	LG	Sheep	20.0	Sandy loam	1.2	8.0	0.2	350	0–60	0.2	+	1.2	7.2	0	3
	Semiarid steppe	C4	LG	Sheep	20.0	Sandy loam	1.2	7.8	0.2	350	0–60	2.0	+	1.2	7.2	0	3
ND (USA)	Mixed grass prairie	M	HG	Steers	20.0	Sandy loam	ND	6.9	ND	384	0–60	0.0	0.0	ND	ND	0	4
	Mixed grass prairie	M	LG	Steers	20.0	Sandy loam	ND	6.9	ND	384	0–60	0.3	27.3	ND	ND	0	4
43°34′N, 119°38′E (CN)	Meadow steppe	C3	MG	Cows	3.0	Clay	1.1	ND	1.0	400	0–30	−	+	ND	ND	0	5
43°34′N, 119°35′E (CN)	Meadow steppe	C3	LG	Cows	3.0	Clay	1.1	ND	1.0	400	0–30	−	+	ND	ND	0	5
43°33′N, 116°40′E (CN)	Steppe grass	C4	HG	Sheep/goats	5.0	Sandy clay loam	1.3	ND	1.0	334	0–30	−	−	1.3	ND	ND	6
	Steppe grass	C4	LG	Sheep/goats	5.0	Sandy clay loam	1.3	ND	1.0	334	0–30	+	+	1.4	ND	ND	6
43°33′N, 116°40′E (CN)	Semiarid steppe	C4	LG	Sheep	30.0	Sandy loam	1.0	6.7	0.7	330	0–10	0.0	−9.5	1.1	6.7	0	7
43°33′N, 116°40′E (CN)	Semiarid steppe	C4	MG	Sheep	30.0	Sandy loam	1.0	6.7	0.7	330	0–10	−0.2	−19.0	1.2	6.7	0	7
43°33′N, 116°40′E (CN)	Semiarid steppe	C4	HG	Sheep	30.0	Sandy loam	1.0	6.7	0.7	330	0–10	−0.4	−40.0	1.3	6.6	0	7
35°57′N, 104°09′E (CN)	Grassland	C4	MG	Sheep	3.0	Sandy soil	1.2	8.4	6.7	382	0–10	−11.3	ND	1.2	8.4	0	8
ND (USA)	Mixed grass prairie	M	LG	Steers	11.0	Sandy loam	1.3	ND	ND	338	0–30	+	+	1.3	ND	ND	9
ND (USA)	Mixed grass prairie	M	MG	Steers	11.0	Sandy loam	1.3	ND	ND	338	0–30	+	+	1.3	ND	ND	9
	Mixed grass prairie	M	MG	Steers	11.0	Sandy loam	1.3	ND	ND	338	0–30	+	+	1.3	ND	ND	9
	Mixed grass prairie	M	HG	Steers	11.0	Sandy loam	1.3	ND	ND	338	0–30	+	+	1.4	ND	ND	9
51°00′N, 112°00′W (CA)	Mixed grass prairie	M	SG	Cattle	26.0	Coarse loam	ND	8.2	4.0	355	0–8	−	ND	ND	ND	ND	10
53°00′N, 111°00′W (CA)	Parkland fescue	C4	SG	Cattle	17.0	Fine loam	2.0	8.2	4.0	422	0–15	−	ND	ND	ND	ND	10
50°00′N, 114°00′W (CA)	Foothills fescue grass	C4	SG	Cattle	41.0	Fine loam	5.0	8.2	4.0	550	0–15	−	ND	ND	ND	ND	10
43°33′N, 116°40′E (CN)	Semi-arid grasses	C4	LG	Livestock	10.0	Loamy sand	1.4	ND	1.0	334	0–50	−1.4	94.0	1.4	ND	0	11
	Semi-arid grasses	C4	HG	Livestock	10.0	Loamy sand	1.4	ND	1.0	334	0–50	−3.8	65.0	1.4	ND	0	11
38°51′N, 105°50′E (CN)	Desert steppe	C4	HG	Livestock	7.0	Sandy soil	1.3	8.4	8.0	210	0–40	1.0	−	1.2	8.1	0	12
36°13′–36°19′N (CN)	Semi-arid grass	C4	ND	Goats	ND	Sandy soil	ND	ND	6.9	425	0–80	−0.5	0.1	ND	ND	ND	13
106°24′–106°28’E (CN)	Semi-arid grass	C4	ND	Goats	ND	Sandy loam	ND	ND	6.9	425	0–80	−0.5	0.1	ND	ND	ND	13
ND (USA)	Short grass steppe	C4	LG	Sheep	12.0	Sandy loam	1.1	ND	ND	366	0–60	0.4	ND	ND	ND	0	14
	Short grass steppe	C4	HG	Sheep	12.0	Sandy loam	1.0	ND	ND	366	0–60	1.3	ND	ND	ND	0	14
	Short grass steppe	C4	LG	Sheep	55.0	Loamy soil	1.1	ND	ND	325	0–60	0.3	ND	ND	ND	0	14
	Short grass steppe	C4	HG	Sheep	55.0	Loamy soil	1.0	ND	ND	325	0–60	1.2	ND	ND	ND	0	14
ND (USA)	Short grass steppe	C4	LG	Livestock	56.0	Loamy soil	1.2	ND	ND	325	0–90	3.1	+	1.2	ND	0	15
	Short grass steppe	C4	HG	Livestock	56.0	Loamy soil	1.2	ND	ND	325	0–90	12.7	+	1.2	ND	0	15
43°38′N, 116°42′E (CN)	Perennial grass	C3	LG	Sheep/goats	4.0	Fine sand	ND	ND	0.7	335	0–5	−	−	ND	ND	0	16
	Perennial grass	C3	MG	Sheep/goats	4.0	Fine sand	ND	ND	0.7	335	0–5	−	−	ND	ND	0	16
	Perennial grass	C3	HG	Sheep/goats	4.0	Fine sand	ND	ND	0.7	335	0–5	−	−	ND	ND	0	16
43°38′N, 116°42′E (CN)	Semiarid steppe	C4	HG	Sheep/goats	30.0	Sandy loam	1.3	6.7	0.7	343	0–4	−	−	1.3	6.6	0	17
43°37′N, 116°41′E (CN	Steppe vegetation	C4	HG	Livestock		Sandy soil	ND	ND	ND	ND	0–50	−	−	ND	ND	ND	18
43°26′–44°08’N (CN)	Temperate grass	C3	HG	Livestock	20.0	Loam/sandy loam	ND	ND	1.1	345	0–40	−1.9	−0.1	ND	ND	ND	19
116°04′–117°05’E (CN)	Temperate grass	C3	HG	Livestock	20.0	Loam/sandy loam	ND	ND	1.1	345	0–40	−1.9	−0.1	ND	ND	ND	19
41°46′N, 115°41′E (CN)	Semi-arid grasses	C4	HG	Sheep/goats/cattle	10.0	Sandy clay loam	1.4	7.6	1.5	350	0–50	−3.9	−0.5	1.5	7.6	0	20
43°38′N, 116°42′E (CN)	Semi-arid grassland	C4	SG	Sheep/goats	25.0	Sandy loam	0.9	ND	0.7	343	0–6	−	ND	ND	ND	ND	21
42°55′N, 120°42′E (CN)	Grass/forbs/shrubs	C4	HG	Cattle/sheep	5.0	Sandy soil	ND	6.4	ND	360	0–20	−	−	ND	ND	0	22

MAAT – mean annual air temperature (°C) and MAP – mean annual precipitation. Changes in SOC (ΔSOC) and total nitrogen (ΔTN) were calculated at 0–30 cm depth using the original depth in each paper and converted into k gm^−2^ of C or N, respectively. ^a^ = Different methods were used to measure soil pH using pH probe/meter in deionized water or 0.01 M CaCl_2_ in 1:1 and 1:2, or 1:5 (v: v) soils: solution ratios. Added N fertilizer is in kg N ha^−1^; OD = original measurements depth; BD = initial bulk density; fBD = bulk density after grazing; ipH = initial pH; fpH = pH after grazing; HG = high grazing; MG = medium grazing; LG = low grazing; SG = grazing series; ND = no data; negative sign = decreased; positive sign = increased; N = added nitrogen fertilizer in kg N ha^−1^. SOC = soil organic carbon; ΔSOC = difference in soil organic carbon between un-grazed and grazed site; ΔTN = difference in total nitrogen between un-grazed and grazed site. C3 = C3 crop; C4 = C4 crop and M = mixed C3/C4 crops. USA = United States of America; CN = China; CA = Canada. Ref = reference: 1 = Barger et al. (2004); 2 = Cao et al. (2013); 3 = Cui et al. (2005); 4 = Ganjegunte et al. (2005); 5 = Han et al. (2008); 6 =  He et al. (2011); 7 =   Kölbl et al. (2011); 8 = Li et al. (2015); 9 = Manley et al. (1995); 10 = Naeth et al. (1991); 11 = Nianpeng et al. (2012); 12 = Niu et al. (2011); 13 =  Qiu et al. (2013); 14 = Reeder and Schuman (2002); 15 =    Reeder et al. (2004); 16 = Schonbach et al. (2012); 17 = Steffens et al. (2008); 18 = Wang et al. (2014); 19 = Wu et al. (2008); 20 = Xu et al. (2014); 21 = Zhao et al. (2007); 22 = Zuo et al. (2008).

**Table 4:** Published studies on the impacts of grazing on SOC in the dry/warm climatic zone.

Coordinates (country/state)	Grass type	C3/C4/M	Grazing intensity	Type of animal	Duration (year)	Soil texture	iBD (g cm^−3^)	ipH^a^	MAAT (°C)	MAP (mm)	OD (cm)	ΔSOC kg m^−2^ C (0–30 cm)	ΔTN kg m^−2^ N (0–30 cm)	fBD (g cm^−3^)	fpH		Ref
MS (USA)	Grass/shrubs/forbs	C4	HG*	Livestock	30.0	Sandy/coarse loam	ND	ND	11.5	207	0–10	−0.4	0.0	ND	ND	ND	1
	Grass/shrubs/forbs	C4	HG*	Livestock	30.0	Fine sandy loam	ND	ND	11.5	207	0–10	−0.4	0.0	ND	ND	ND	1
	Grass/shrubs/forbs	C4	HG*	Livestock	30.0	Coarse loamy soil	ND	ND	11.5	207	0–10	−0.3	0.0	ND	ND	ND	1
54°02′–54°15′E; 37°10′–37°18′N (IR)	Grass/brushes	C3	HG	Livestock	27.0	Silty loam	ND	ND	17.0	343	ND	−	ND	ND	ND	ND	2
41°03′S, 70°31′W (AR)	Wet meadow	C4	HG	Sheep	20.0	Peat soil	1.0	6.6	8.3	280	0–100	−	ND	1.2	6.8	0	3
	Mesic meadow	C4	HG	Sheep	20.0	Peat soil	1.1	7.9	8.3	280	0–100	−	ND	1.3	8.8	0	3
	Wet meadow	C4	HG	Sheep	20.0	Sandy loam	1.3	8.7	8.3	150	0–100	−	ND	1.3	9.3	0	3
44°28′N, 38°56′E (IR)	Grassy rangeland	C3	HG	ND	ND	ND	ND	ND	12.0	265	0–30	+	ND	ND	ND	ND	4
45°51′N, 70°16′W (AR)	Grass steppe/shrubs	C4	MG	Sheep	ND	Sandy clay	ND	ND	ND	150	0–5	0.0	ND	ND	ND	ND	5
	Grass steppe/shrubs	C4	HG	Sheep	ND	Sandy clay	ND	ND	ND	150	0–5	0.0	ND	ND	ND	ND	5
41°11′N, 104°53′W (USA)	Mixed grass prairie	M	LG	Cattle	10.0	Fine loamy	1.3	6.9	13.0	425	0–60	1.5	0.1	ND	ND	ND	6
	Mixed grass prairie	M	HG	Cattle	10.0	Fine loamy	1.3	6.9	13.0	425	0–60	−1.2	−0.1	ND	ND	ND	6
42°27′S, 64°34′W (AR)	Perennial grass/shrubs/herbs	C4	HG	Sheep	100.0	Silty soil	1.1	ND	13.0	188	0–30	−	ND	1.2	ND	ND	7
41°47′N, 111°53′E (USA)	Desert steppe	C4	LG	Sheep	ND	Loamy sand	1.3	7.5	3.4	280	0–30	−0.6	0.0	ND	ND	ND	8
	Desert steppe	C4	MG	Sheep	ND	Loamy sand	1.3	7.5	3.4	280	0–30	−0.7	0.0	ND	ND	ND	8
	Desert steppe	C4	HG	Sheep	ND	Loamy sand	1.3	7.5	3.4	280	0–30	−0.6	0.0	ND	ND	ND	8
43°38′N, 116°42′E (USA)	Typical steppe	C4	LG	Sheep	ND	Fine sand	1.2	7.7	0.7	335	0–30	1.0	0.0	ND	ND	ND	8
	Typical steppe	C4	MG	Sheep	ND	Fine sand	1.2	7.7	0.7	335	0–30	0.2	0.0	ND	ND	ND	8
	Typical steppe	C4	HG	Sheep	ND	Fine sand	1.2	7.7	0.7	335	0–30	0.8	0.0	ND	ND	ND	8
41°46′N, 111°02′E & 41°46′N, 111°53′E & 41°50′N, 111°55′E (CN)	Desert steppe	C4	LG	Sheep	30.0	Loamy sand	1.4	7.9	ND	280	0–20	0.0	0.0	1.4	7.9	ND	9
	Desert steppe	C4	MG	Sheep	30.0	Loamy sand	1.4	7.9	ND	280	0–20	−0.6	0.0	1.3	8.0	ND	9
	Desert steppe	C4	HG	Sheep	30.0	Loamy sand	1.4	7.9	ND	280	0–20	−0.3	0.0	1.4	8.0	ND	9
21°49′N, 101°37′W (MX)	Short grass steppe	C4	MG	Livestock	200.0	Silty clay/sandy clay	ND	ND	18.0	380	0–30	+	−	ND	ND	ND	10
	Short grass steppe	C4	HG	Livestock	200.0	Silty clay/sandy clay	ND	ND	18.0	380	0–30	−	−	ND	ND	ND	10
ND (USA)	Grass/shrubs	C4	HG	Cattle	ND	Fine sandy loam/fine sand Coarse sand	1.4	ND	ND	270	0–50	−0.4	ND	1.4	ND	ND	11
	Grass/shrubs	C4	HG	Cattle	ND	Fine sandy loam/fine sand Coarse sand	1.4	ND	ND	270	0–50	−0.3	ND	1.4	ND	ND	11
42°06′S, 71°10′W (AR)	Grass-shrub steppe	C4	HG	Livestock	ND	Sandy soil	ND	6.0	ND	424	0–200	−0.2	ND	ND	ND	ND	12
39°08′N, 105°35′E (CN)	Grass/shrubs/forbs	C4	HG	Sheep	6.0	Sandy soil	1.5	9.0	9.1	174	0–40	−0.3	ND	1.6	9.0	ND	13
	Grass/shrubs/forbs	C4	HG	Sheep	2.0	Sandy soil	1.5	9.0	9.1	174	0–40	−0.1	ND	1.6	9.0	ND	13
31°50′N, 51°14′E (IR)	Rangeland	C3	HG	Sheep/goats	0.5	Silty clay	1.6	7.5	10.7	225	0–15	0.1	0.0	1.7	7.6	ND	14
ND (USA)	Mixed-grass prairie	M	MG	Cattle	12.0	Fine loamy	0.9	ND	21.0	458	0–20	−2.4	ND	ND	ND	ND	15
	Mixed-grass prairie	M	HG	Cattle	12.0	Fine loamy	0.9	ND	21.0	458	0–20	−2.2	ND	ND	ND	ND	15
ND (USA)	Mixed grass prairie	M	LG	Steers	12.0	Sandy loam	1.4	6.9	6.0	384	0–60	0.5	0.1	1.3	ND	ND	16
	Mixed grass prairie	M	HG	Steers	12.0	Sandy loam	1.4	6.9	6.0	384	0–60	1.6	0.0	1.5	ND	ND	16
41°11′N, 104°54′W (USA)	Grass/fobs/sedge	M	LG	Steers	12.0	Sandy loam	1.4	6.9	7.5	384	0–60	−0.1	ND	ND	ND	0	17
	Grass/fobs/sedge	M	HG	Steers	12.0	Sandy loam	1.4	6.9	7.5	384	0–60	−0.3	ND	ND	ND	0	17
24°45′N, 31°22′E (ZA)	Grassy shrubland	C4	LG	Sheep/goats	75.0	Sandy clay silt	1.6	6.5	14.4	373	0–60	0.0	0.0	1.6	7.3	0	18
	Grassy shrubland	C4	HG	Sheep/goats	75.0	Sandy clay silt	1.6	6.5	14.4	373	0–60	0.0	0.0	1.7	7.1	0	18
25°56′S, 22°25′E (BW)	Grass/woody shrubs	C4	MG	Livestock	ND	Sandy soil	ND	7.0	6.1	331	0–100	−	ND	ND	ND	0	19
42°58′N, 120°43′E (CN)	Grass/shrubs/forbs	C4	MG	Cattle/sheep	ND	Sandy soil	1.44.0	7.9	6.5	366	0–15	0.1	0.0	4.8	4.6	0	20
	Grass/shrubs/forbs	M	MG	Cattle/sheep	ND	Sandy soil	1.44.0	7.9	6.5	366	0–15	0.0	0.0	4.8	4.6	0	20

MAAT – mean annual air temperature (°C) and MAP – mean annual precipitation. Changes in SOC (ΔSOC) and total nitrogen (ΔTN) were calculated at 0–30 cm depth using the original depth in each paper and converted into kg m^−2^ of C or N, respectively. ^a^ = Different methods were used to measure soil pH using pH probe/meter in deionized water or 0.01 M CaCl_2_ in 1:1 and 1:2, or 1:5 (v: v) soils: solution ratios. Added N fertilizer is in kg N ha^−1^. OD = original measurements depth. BD = initial bulk density; fBD = bulk density after grazing; ipH = initial pH; fpH = pH after grazing; ^S^ = simulation study; HG = high grazing; MG = mdium grazing; LG = low grazing; * = originally described as free grazing; MG^+^ = originally described as national grazing; ND = no data; negative sign = decreased; positive sign = increased; N = added nitrogen fertilizer in kg N ha^−1^. SOC = soil organic carbon; ΔSOC = difference in soil organic carbon between un-grazed and grazed site; ΔTN = difference in total nitrogen between un-grazed and grazed site. C3 = C3 crop; C4 = C4 crop and M = mixed C3/C4 crops. Ref = reference: 1 =    Fernandez et al. (2008); 2 = Asgharnezhad et al. (2013); 3 = Enriquez et al. (2015); 4 =   Ghoreyshi et al. (2013); 5 = Golluscio et al. (2009); 6 =  Ingram et al. (2008); 7 = Larreguy et al. (2014); 8 = Liu et al. (2012); 9 = Li et al. (2008); 10 =  Medina-Roldana et al. (2008); 11 = Neff et al. (2005); 12 = Nosetto et al. (2006); 13 =  Pei et al. (2008); 14 = Raiesi and Riahi (2014); 15 = Rogers et al. (2005); 16 = Schuman et al. (1999); 17 = Schuman et al., 2002, Schuman et al., 2009; 18 = Talore et al. (2016); 19 = Thomas (2012); 20 =  Su et al. (2005). IR = Iran; USA = United States of America; AR = Argentina; CN = China; MX = Mexico; BW = Botswana; ZA = South Africa.

### 2.2 Estimation methods applied

In some studies SOC and TN values are given as concentrations. To convert these values to stocks (kg m^−2^), the following equations were applied (IGBP-DIS, 1998):

(1) SOC (kg m^−2^) = [depth (cm) × BD (g cm^−3^) × SOC (%C in g per100 g soil)]/1000

(2) TN (kg m^−2^) = [depth (cm) × BD (g cm^−3^) × TN (%TN in g per100 g soil)]/1000

In cases where there were more than one year of values reported in the original paper we used the mean value in this meta-analysis. However, because studies reported the SOC and TN content from different soil depths, we used a quadratic density function based on Smith et al. (2000) to derive a scaling cumulative distribution function (c.d.f.) for soil density as a function of soil depth up to 1m. This allows SOC and TN at a given depth d (m) to be scaled to the equivalent values at 0.30 m as follows:

(3) $$\text{cdf}\left(\text{d}\right)=\left(22.1-\frac{33.3{\text{d}}^{2}}{2}+\frac{14.9{\text{d}}^{3}}{3}\right)/10.41667$$

(4) SOC(0.3m)=SOC(d)×(cdf(0.3))/(cdf(d))

Different methods were used to measure soil pH in different studies, e.g. using pH probe/meter in deionized water or 0.01 M CaCl_2_ in 1:1 and 1:2 or 1:5 (v:v) soils: solution ratios. We did not adjust pH results recorded by different methods, but where a range of values were reported, we took the mean value. Also, where a range of air temperatures was reported, we used mean annual value in degree Celsius (°C) as reported for the years of the study in the meta-analysis. The mean annual precipitation (mm) value for each study period was taken from the original papers. However, where the mean annual precipitation or mean annual temperature were not reported, those values were taken from the CRU 3.24 climate data set (Harris et al., 2013).

The GI reported in each of the studies was estimated in different ways, and was usually subjective, depending on local practices, and usually described as high, medium (or moderate) and low. To undertake this analysis we required a continuous variable for grazing intensity and so the method described below was developed for this study and used to classify the GI used for each of the experiments in a comparable way. As available forage was not described in all studies it was necessary to estimate the amount of plant dry material available (DM) on each site annually and to calculate the forage requirements for the animals grazed at each experimental plot in a consistent manner. To achieve this, the annual NPP, expressed as dry vegetable matter (DM) (mg DM ha^−1^ y^−1^) in terms of C was predicted for each location using the Miami model (Leith, 1972, Grieser et al., 2006) and calculated using mean annual precipitation (P, in mm), and mean annual temperature (T, in °C) reported in each study or determined from the CRU TS 3.4 dataset (i.e. possible effect of N fertilizer was not considered because of data scarcity however; N application rates would generally be considered low in extensively grazed systems).

(5) NPP = minimum (NPP_T_; NPP_P_)

(6) NPP_T_ = 30 (1 + exp (1.315 *− *0.119 T)

(7) NPP_p_ = 30 (1 − exp (−0.000664 P))

where NPP_T_ is the net primary production calculated based upon temperature and NPP_p_ is the net primary production calculated based upon precipitation (Leith, 1972, Grieser et al., 2006).

The available surface vegetable dry matter (SVDM) available for animal grazing for each location was calculated using the following relationship, assuming an allocation of NPP to above ground biomass of 50% (Li et al., 1994):

(8) SVDM = NPP × 0.5 (mg DM ha^−1^ y^−1^)

An animal unit month (AUM) is considered as a bovine weighing of 500 kg requiring 350 kg of DM a month of feed, based on the animal equivalent chart (USDA-Animal equivalent chart, USDA, 2017). The carrying capacity (CC) of grassland is the number of animal unit months that the land will support, based upon the available forage dry matter and the energy requirement, and this we calculated as:

(9) CC = SVDM/0.350 AUM ha^−1^ y^−1^

The GI was calculated from the ratio of the number of animal unit months actually grazed up to carrying capacity. The actual number of animal unit months (AAUM) depended on the type of animal: i) cows = 1; ii) steers = 0.7; iii) sheep = 0.2; iv) goats = 0.2, v) domesticated yaks = 0.7 (USDA-Animal equivalent chart, USDA, 2017). The AAUM was calculated as the product of stocking density per ha multiplied by the number of months grazed per year in ha^−1^ y^−1^.

(10) GI = AAUM/CC

As changes in SOC stocks are related to the initial SOC and the annual carbon input to the soil. We calculated the annual carbon input (CIN) to be the quantity of annual NPP carbon not grazed by the animals, and calculated as:

(11) CIN = NPP (1 − GI).

### 2.3 Data analyses

We used Minitab 17 (Minitab, Inc., State College, PA) to conduct the data exploration, conditioning and analyses. The complete data set was analysed to estimate the overall impact of grazing on grassland SOC and selected soil properties, and then to analyse the impact of climatic zone and GI. We have sufficient data to estimate the change in SOC stock (n = 83) related to grazing for the top 30 cm or the profile over the period of the experiment that could be normalized to an annual rate per year. For a subset of the data (n = 64), it was possible to estimate the change in total nitrogen per year during the experiment, bulk density change (n = 43), and pH (n = 30).

The data collected were segregated into four climatic zones for the meta-analysis: DC (n = 26), DW (n = 33), MC (n = 9) and MW (n = 15). The data were also grouped by the calculated GI: low (LG; GI = 0–0.33), medium (MG; GI = 0.33–0.66), high (HG; GI = 0.66–1.0) and overgrazed (OG; GH ≤ 1.0). The tests were also grouped by animal type bovine (B), which included yaks, steers, cows and heifers; caprine (C), including sheep and goats; and a mixture of both bovine and caprine (M). The tests were also grouped by soil type and texture: clay, clay-loam, loam, sandy-loam and sandy; and grassland type: grassland, shrubby grassland, woody grassland, steppe, and prairie. We also tested grass by photosynthetic pathway type: C3, C4 and mixed.

We used different analytical procedures for each group and parameter that related to the available published data. An analysis of the effects of grazing on SOC, TN, pH and BD was made by the methods of Hedges et al. (1999) and Luo et al. (2006) using the response ratio (RR) defined as the natural logarithm of the ratio of the value or the parameter measured on the grazing treatment to that without grazing (control).

(12) Ln (RR) = ln (grazed treatment parameter value/un-grazed (control) parameter value)

The rate of change (R) was calculated in the form ln (RR) by dividing by the length of the experiment in years (y).

(13) R = ln (RR)/y

The descriptive statistics of the annual change in SOC, TN, BD and pH due to grazing including mean, median, standard deviation, and 95% confidence intervals for each were calculated. One way ANOVAs were performed to investigate the impact of factors: climate, GI, grass and animal types on SOC, TN and other selected soil properties, and the rates of change. Principle component analysis was used to determine significant explanatory variables and response variables and determine the differences between climatic zones. In addition, regressions or mixed models such as GLM's, were used to determine significant explanatory variables.

## 3 Results

### 3.1 Estimation of NPP and grazing intensities

**Fig. 2:**
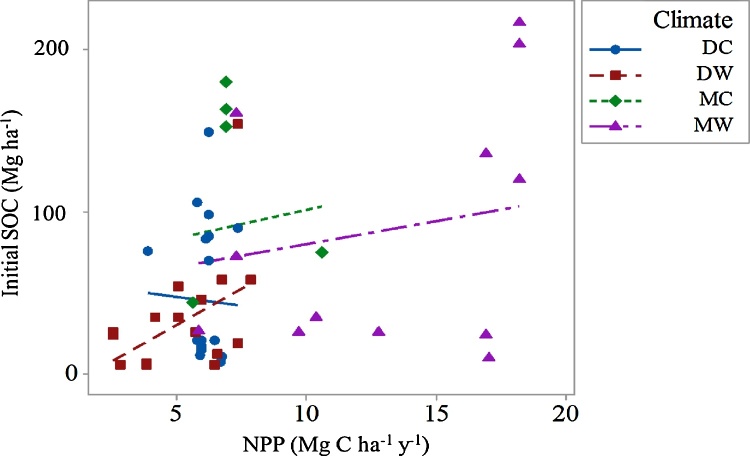
The initial SOC (mg ha^−1^) and NPP values (mg mg C ha^−1^ y^−1^) for different climatic zones (DC = dry cool, DW = dry warm, MC = moist cool, MW = moist warm), 0–30 cm depth.

**Fig. 3:**
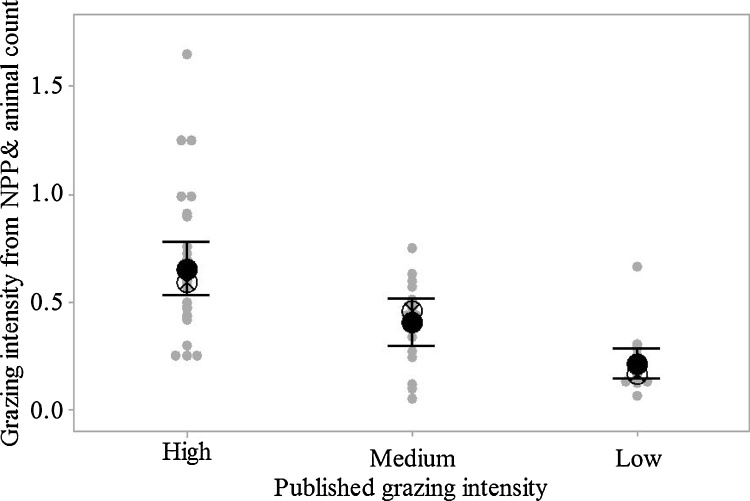
Comparison of published grazing intensities (high, medium and low) compared with those derived from NPP and number of animals. The symbols are showing the median (⊗) and the mean (●), with 95% confidence interval as a bar and individual site values as grey dots.

Mean NPP for the period 1960–2000 covered a wide range of values reflecting the global diversity of NPP under different climatic zones (Fig. 1). In addition to decomposition rates, SOC content partly depends on OC input. No statistically significant differences in NPP between the DC, DM and MC climatic zones was found; however, the NPP values at the MW climate were significantly greater from those under the other climatic zones (Fig. 2 and Table 5). The calculated and reported estimates of GIs show considerable overlap, and only three experiments represented ‘overgrazing’ i.e. beyond the carrying capacity of the system (Fig. 3). They also illustrated the different definitions of the levels of grazing used in the literature for each domain.

**Table 5:** Comparison of NPP by climatic zones (p < 0.001).

Climatic zone	N	Mean Stdev. (mg C ha^−1^ y^−1^)		95% CI	Grouping Tukey
Dry cool	26	6.0	0.7	(5.0, 6.9)	B
Dry warm	33	5.4	1.6	(4.5, 6.2)	B
Moist cool	9.0	7.2	2.1	(5.5, 8.7)	B
Moist warm	15	12.7	4.9	(11.4, 13.9)	A

**Fig. 4:**
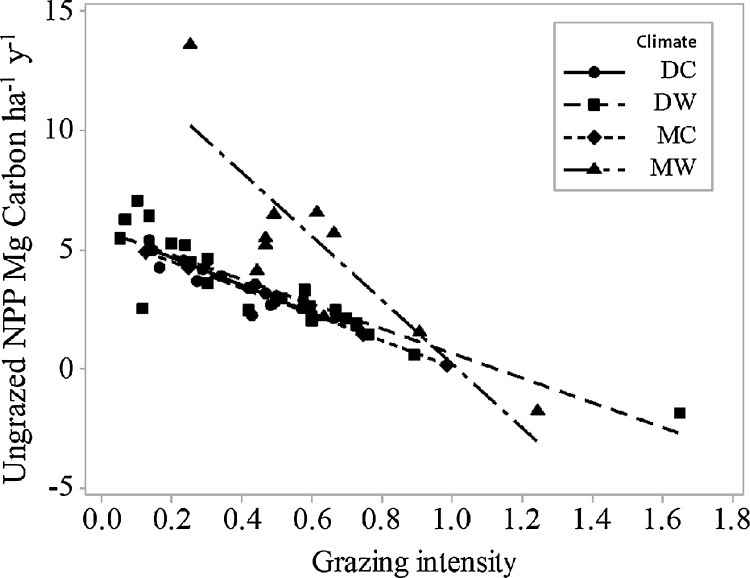
Regression of un-grazed NPP (mg C ha ^−1^ y^−1^) to grazing intensity calculated from NPP and number of animal units (values greater than zero are overgrazed) for each climatic zone (DC = dry cool, DW = dry warm, MC = moist cool, MW = moist warm).

A linear regression of annual NPP remaining available as a possible OC input to the soil, with the calculated GI and climatic zones (p < 0.001, R^2^ = 67%), demonstrated that the SOC stock under the MC climatic zone is much higher than under the other climatic zones (Fig. 4). The second highest climatic zone, in SOC, is MW but with much higher standard deviation (data not shown). An ANOVA showed that un-grazed SOC is different between the different climatic zones as shown in Table 6 and explains 21% of the variation. A GLM showed that adding NPP and pH explained 41% of the un-grazed SOC value.

**Table 6:** Comparison of non-grazed SOC by climatic zones (p < 0.001).

Climatic zone	N	Mean Stdev. (mg C ha^−1^ y^−1^)		95% CI	Grouping Tukey
Dry cool	26	45.2	40.3	(27.1, 62.3)	BC
Dry warm	33	34.0	29.8	(18.0, 50.0)	C
Moist cool	9.0	91.2	57.2	(60.6, 121.8)	AB
Moist warm	15	87.2	72.2	(63.5, 110.9)	A

### 3.2 Impacts of grazing intensity on SOC and other selected soil properties using the response ratio ln (RR)

**Fig. 5:**
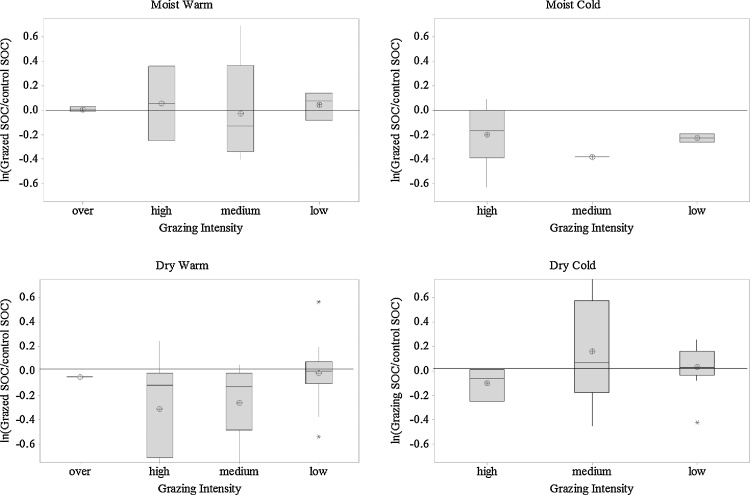
Impacts of grazing on soil organic carbon (SOC) stocks (0–30 cm soil depth) under the different climatic zones. (DC = dry cool, DW = dry warm, MC = moist cool, MW = moist warm). Grazing intensities are described as percentage of the annual net primary production (over (grazed) ≥ 100%, high = 100–66%, medium = 66–33%, low ≤ 33%). Impact in the natural logarithm of the ratio of un-grazed SOC to grazed SOC. ⊕ is mean, box shows 95% confidence and median as a bar.

An analysis of all studies together and using the response ratio ln (RR) of grazed compared to un-grazed grassland, showed that GI was associated with a decrease of overall SOC stocks by a response ratio of −0.0774 (−8%; StDev = 0.358). It was also associated with a slight increase in pH of 0.029 (+3%; StDev = 0.044), an increase in TN of 0.06 (+6%; StDev = 0.772) and BD of 0.070 (+7%; StDev = 0.083). However, an ANOVA of the SOC, TN, BD and pH showed that whilst climatic zone significantly affects SOC change (p = 0.011) and pH (p = 0.014), it did not significantly impact BD (p = 0.144) or TN (p = 0.118) (Table 7). At all GI levels, grazing increased SOC stocks under the MW climate (+7.6%), but decreased them under the MC climate (−19.5%). However, for the DW and DC climates, only the low (+5.8%) and low to medium (+16.1%) grazing intensities, respectively, led to increases in SOC (Fig. 5).

**Table 7:** Natural logarithm of response ratio effects for SOC, TN, pH and BD by climatic zones. N = number of studies.

ln (RR) function	Climatic zone	N	Mean Stdev. ln (treatment/control)		95% CI	Grouping Tukey
SOC (P = 0.011)	Dry cool	26	0.076	0.316	(−0.056, 0.209)	A
	Dry warm	33	−0.195	0.392	(−0.312, −0.076)	B
	Moist cool	9	−0.227	0.209	(−0.453, −0.001)	AB
	Moist warm	15	0.004	0.316	(−0.170, 0.179)	AB
Total N (P = 0.118)	Dry cool	7	0.233	0.317	(−0.335, 0.801)	A
	Dry warm	21	−0.119	0.284	(−0.446, 0.209)	A
	Moist cool	5	−0.124	0.184	(−0.796, 0.548)	A
	Moist warm	5	0.754	2.014	(0.082, 1.425)	A
Bulk density (P = 0.014)	Dry cool	9	0.000	0.015	(−0.026, 0.026)	B
	Dry warm	11	0.056	0.054	(0.032, 0.080)	A
	Moist cool	9	0.019	0.029	(−0.007, 0.044)	AB
	Moist warm	1	0.072	n/a	n/a	AB
pH (P = 0.144)	Dry cool	15	0.076	0.074	(0.034, 0.117)	A
	Dry warm	13	0.045	0.066	(0.000, 0.089)	A
	Moist cool	9	0.117	0.111	(0.062, 0.179)	A
	Moist warm	4	0.025	0.054	(−0.056, 0.105)	A

Analysis of the impact of animal type (bovine, caprine and mixed) on ln (RR) of SOC across all climate types showed no significant difference (p = 0.89). Neither soil texture (clay, clay-loam, loam, sandy-loam and sandy) (p = 0.75), nor grassland characteristics (grassland, shrubby grassland, woody grassland, steppe, and prairie) (p = 0.079) significantly affected SOC levels. However, an ANOVA for grass photosynthetic pathway type (C3, C4 and mixed) showed that there was a significant difference (p = 0.003) with C4 grasslands increasing SOC by 0.056 (5.6%; StDev = 0.341), and C3 grasses and mixed grass decreasing SOC by −0.155 (−15.5%; StDev = 0.233) and −0.25 (−25%; StDev = 0.435), respectively (Table 8).

**Table 8:** Natural logarithm of response ratio effects for SOC by grass type.

Climatic zone	Grass type	N	Mean Stdev. ln (treatment/control)		95% CI	Grouping Tukey
SOC (P = 0.003)	C3	25	−0.155	0.233	(−0.289, −0.020)	B
	C4	39	−0.056	0.341	(−0.051, 0.163)	A
	M	19	−0.250	0.435	(−0.304, −0.095)	B

### 3.3 Impacts of grazing intensity on SOC with annual rate of response ratio ln (RR)

The annual rate of change, R, of the response ratio ln (RR), show that overall GI decreased SOC, with an annual rate of −0.009 (−0.9%; StDev = 0.037), but increased pH at a rate of 0.003 (+0.3%; StDev = 0.006), TN at a rate of 0.0005 (+0.05%; StDev = 0.0047) and BD at a rate of 0.009 (+0.09%; StDev = 0.021). However an ANOVA of the SOC, TN, BD and pH showed that, whilst climatic zone significantly impacts the rate of SOC change (p < 0.001), rate of TN (p = 0.047) and rate of BD change (p = 0.009), it did not significantly impact the rate of pH change (p = 0.201) (Table 9). It also showed that GI was associated with more rapid decreases in SOC in DW and MC climates, than in DC and MW climates (Table 9).

**Table 9:** Natural logarithm of response ratio effects for SOC, TN, pH and BD by climatic zone. N = number of studies.

ln (RR) function	Climatic zone	N	Mean Stdev. ln (treatment/control)		95% CI	Grouping Tukey
SOC (P < 0.001)	Dry cool	26	0.002	0.020	(−0.010, 0.014)	A
	Dry warm	33	−0.016	0.032	(−0.030, 0.000)	A
	Moist cool	9	−0.057	0.057	(−0.077, −0.035)	A
	Moist warm	15	0.007	0.027	(−0.009, 0.022)	B
Total N (P = 0.047)	Dry cool	7	0.017	0.022	(−0.001, 0.035)	A
	Dry warm	21	−0.005	0.013	(−0.019, 0.008)	A
	Moist cool	5	−0.019	0.040	(−0.040, 0.003)	A
	Moist warm	5	0.013	0.026	(−0.009, 0.034)	A
Bulk density (P = 0.009)	Dry cool	9	0.004	0.004	(−0.005, 0.013)	B
	Dry warm	11	0.004	0.008	(−0.007, 0.015)	B
	Moist cool	9	0.029	0.036	(0.017, 0.041)	A
	Moist warm	1	0.000	0.001	(−0.018, 0.018)	AB
pH (P = 0.201)	Dry cool	15	0.000	0.001	(−0.004, 0.003)	A
	Dry warm	13	0.003	0.005	(−0.001, 0.007)	A
	Moist cool	9	0.006	0.008	(0.001, 0.009)	A
	Moist warm	4	0.003	n/a	(−0.008, 0.014)	A

### 3.4 Interactions between climatic zone, grazing intensity and soils

The effect of soil texture was tested by ANOVA both for the entire data set (n = 67) and for each climatic region (DC, n = 22; DW, n = 21; MC, n = 6 & MW, n = 14), but no statistical differences were found between texture classes (data not shown).

### 3.5 Interactions of significant explanatory variables on response ratio ln (RR)

**Fig. 6:**
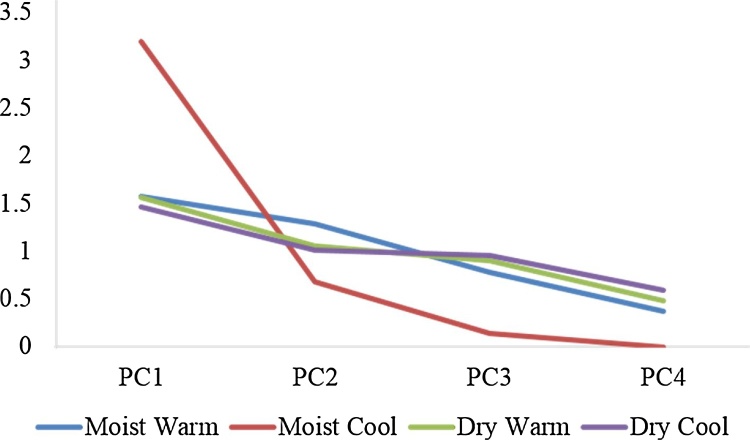
Principle component analysis for four climatic zones using Ln (response ratio soil organic carbon), Initial soil organic carbon to 30 cm, grazing intensity on a scale of 0–1 and net primary productivity (NPP) as variables.

**Fig. 7:**
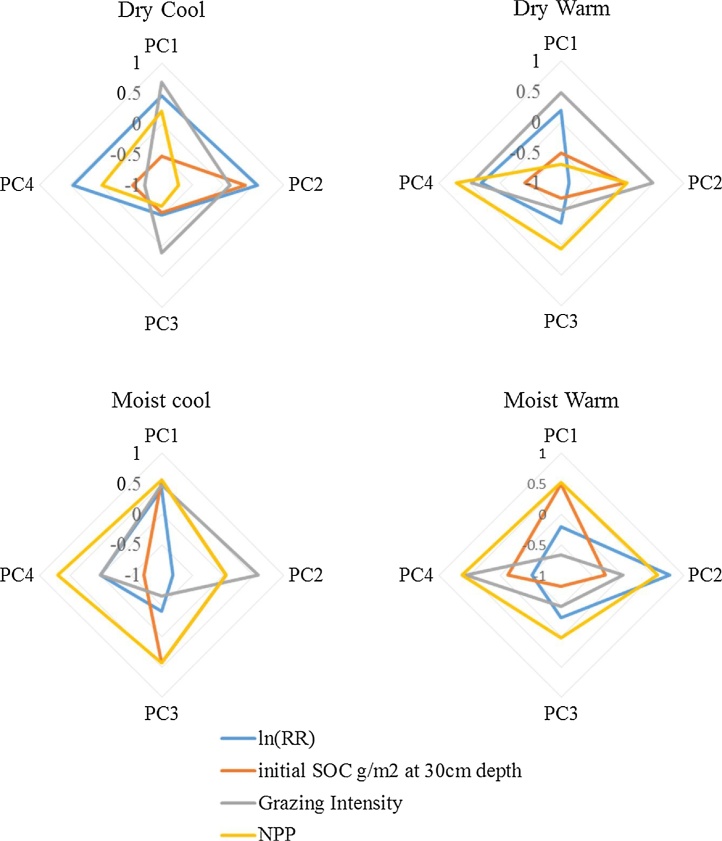
Radar plot of the contribution of explanatory variables: initial soil organic carbon to 30 cm, grazing intensity on a scale of 0–1 and net primary productivity (NPP) and response variable Ln (response ratio soil organic carbon) (ln(RR)) to four principle components for four climatic zones.

Principle component analysis (PCA) showed that the main explanatory variables for response ratio ln (RR) were climatic zone, initial SOC, grazing intensity and NPP. PCA component 1–4 derived from this parameter subset showed a different pattern for each climatic zone with DW and DC being similar and MW and MC exhibiting different patterns (Fig. 6). When the contribution of each variable to the four components is examined in radar plots (Fig. 7), it is observed that the pattern of interaction of each variable is different for each climatic zone indicating that SOC change is governed by different factors.

## 4 Discussion

### 4.1 Comparison of methods used here with previous analyses

In this systematic global review and meta-analysis we collected 83 published studies, on the impacts of GI of grasslands on SOC and other selected soil properties, covering 164 sites and representing different countries and climatic zones. Unlike previous published reviews (e.g. McSherry and Ritchie, 2013, Lu et al., 2017, Zhou et al., 2017), we depth-normalized the SOC and TN data in line with IPCC guidelines. We also calculated a normalized GI, with the aim of harmonising very heterogeneous data. Additionally, the calculation of the normalized GI allowed us to compare across experiments, since reported grazing intensities were subjective, considering the normal local management practices. We found the calculated GI overlapped with the GI from the collected literature, which suggests that our normalization method is unlikely to have introduced additional errors. The extracted mean annual temperatures and annual rainfall at each site from the CRU 3.4 dataset all agreed well with the values reported in publications, where given, providing confidence to the calculation of NPP using the Miami model at each experimental site. Our values of excess NPP for a given GI are similar for all climatic zones except for MW, where the value is almost double that in the other climatic zones. Here climate, especially temperature and rainfall, influences grass productivity and thereby NPP (Chu et al., 2016). Climatic zones also play a major role in the initial SOC contents, and values for the different zones were significantly different (p < 0.05) from each other (i.e. SOC was highest for MC, and lowest for the DW climatic zone). Estimation of uncertainty is of crucial importance since it has a large impact on the management decisions. In this study, some approximations and assumptions incorporated in the methods we used may have created uncertainty in the final results. To consider this, we have conservatively estimated it by calculating the standard deviation for all values as shown in the Table 5, Table 6, Table 7, Table 8, Table 9.

### 4.2 Impacts of grazing intensity on soil organic carbon (SOC)

By pooling all the data and ignoring the regional climatic zones we found that higher GI (below the carrying capacity of the systems), was generally associated with a decrease in SOC stocks. Similar results were found by Lu et al. (2017) and Zhou et al. (2017) among others. The effects of GI management on SOC are mediated by ground cover and high organic matter supply and/or less soil erosion (Waters et al., 2017). High GI can decrease net primary productivity (Wardle, 2002) and result in the loss of palatable, larger-leaved species causing domination of unpalatable small-leaved species which produce litter of low quality for soil microbes and fauna (Cornelissen et al., 1999, Pavlů et al., 2007, Shengjie et al., 2017). This reduction of some plant-species could also result in decreasing chemical quality of the organic C stock (i.e. reducing of water soluble C) in soil (Larreguy et al., 2017). Moreover, high GI can shift the fungal- to- bacterial ratio towards dominance by fungi, which are more tolerant of periodic drought and seasonal fluctuations in soil moisture than bacteria (Bagchi and Ritchie, 2010, Bagchi et al., 2017). In a world of a changing climate livestock production will be negatively affected, especially in arid and semiarid regions, due to e.g. diseases and water availability. High GI under increased frequency of drought and heat wave events may increase GHG emissions and turn grasslands into C sources (Ciais et al., 2005, McSherry and Ritchie, 2013). Additionally, long-term drought in combination with high atmospheric CO_2_ concentration can decrease soil microbial biomass and promotes a shift in functional microbial types, and thereby, modify biogeochemical cycles and SOC storage (Barnard et al., 2006, Pinay et al., 2007).

However, analysing our data according to climatic zone revealed that the impact of GI on SOC is clearly climate dependent, so that the same GI level in different climatic zones could have different impacts on SOC stocks. This can be explained by the interactions between GI and the environmental parameters (e.g. temperature and precipitation) at each climatic zone. The different GI levels have significantly different effects on individual plant species occurrences and covers and thereby, SOC. Generally, grazing stimulates pasture growth, so although the animals under high GI consume more C from the system and respire it, grazing returns (urine and faeces) recycle the C so, the input to the soil remains similar. In addition, the amount and quality of animal urine and dung, and typical manure management practices in each climatic zone, may also stimulate grass regrowth differently. Further, high GI on dry areas or C3 grassland reduces C storage and makes it vulnerable to climate change whilst increases C sequestration under C4 grasslands. Below we discuss our results for each climatic zone in more detail.

#### 4.2.1 Impacts of grazing intensity on soil organic carbon (SOC) under dry/warm climates

Under the DW climate, where soil is dry and temperature and evapotranspiration are high, GI has detrimental effects on SOC at all levels apart from low GI, where SOC increases by 5.8%. In this climatic zone, Angassa (2014) reported a decline in species richness under high GI and suggested low to medium grazing intensities for promoting and conserving key forage species. Low GI could stimulate grass regrowth and mobilise nutrients within the soil and is therefore, recommended for steppe-type ecosystem such as those found in Inner Mongolia (Steffens et al., 2008). Fernandez et al. (2008) reported that high GI decreases soil fertility and has long-term potential implications for the sustainability of grazing in semi-arid environments. It can also increase CO_2_ fluxes from soil and reduce the potential of grasslands to capture CO_2_ by reducing aboveground biomass (Frank et al., 2002), thereby reducing the source of SOC from above- and below-ground inputs. Similarly, in a mixed prairie, high GI has been shown to change grass composition (reduced tallgrasses) resulting in reduced litter accumulation and ground cover (Fuhlendorf et al., 2002). It is also likely to increase nutrient losses (particularly N) (Craine et al., 2009), and affect bacterial and fungal community structures (Huhe Chen et al., 2017); hence threaten longer term sustainability. However, according to Talore et al. (2016), although high GI reduces SOC and TN content and its C/N ratio, a resting period of 1-2 years followed by three consecutive grazing years at low GI would improve SOC and be ideal for sustainable livestock production in South Africa. In addition, Walters et al. (2017) reported that management of GI by rotational grazing (which incorporating long periods of rest) also increased SOC on red Lixisol soils.

#### 4.2.2 Impacts of grazing intensity on soil organic carbon (SOC) under moist/cool climates

In the MC climatic zone, where soil is moist for longer periods and the temperature is low, all grazing led to a decrease in SOC. The activity of soil microorganisms is supressed due to low temperature and high water saturation of the soil (i.e. reducing oxygen availability). High rainfall decreases microbial biomass, possibly due to high demand of nutrients from the soil for the peak growth of vegetation during that time (Devi et al., 2014) and decreases soil pH (Slessarev et al., 2016). Many other studies in MC climates have found that frequent disturbances of grassland by grazing practices at different intensities decrease C sequestration in soils (e.g. Klumpp et al., 2007, Klumpp et al., 2009, Wu et al., 2009, Wu et al., 2010). Sun et al. (2011) reported that higher GI under alpine meadows, reduced plant biomass productivity and changed the species composition and thereby, decreased SOC. Moreover, Wu et al. (2009) and Dong et al. (2012) found that high GI decreased not only SOC, but also soil N in the Qinghai-Tibetan Plateau. Further, trampling by cattle decreases SOC storage by stimulating organic matter decomposition, due to the destruction of soil aggregates by mechanical stress, alters soil microbial community structure, leading to lower fungal- to- bacterial ratios (Hiltbrunner et al., 2012), and increase denitrification rates and N losses (Su et al., 2005; Jones et al., 2016). Pappas and Koukoura (2011) found that medium GI could enhance soil C accumulation at higher altitudes. The trade-off between above- and belowground C storage is positively associated with net ecosystem productivity. However, increasing grass productivity by adding more N fertilizer then intensifying the GI accordingly can increase SOC (Klumpp et al., 2007). Although the use of added inorganic N fertilizer to enhance productivity in temperate grasslands is widespread, it can lead to an enhancement of N losses particularly as GI increases. This can lead to a situation where despite increases in C sequestration the losses of non CO_2_ GHGs (e.g. N_2_O) increase and the net GHG balance remains close to zero (or becomes positive), offsetting the benefits of C sequestration (Jones et al., 2016, Soussana et al., 2007). In circumstances where soils have a high nutrient capital (e.g. upland sheep grazing), it can be more appropriate to recommend no or low-intensity grazing as a management practice for enhancing plant and soil C sequestration (Smith et al., 2014). In contrast, Gao et al., 2007, Gao et al., 2009 and Li et al. (2011) reported that higher GI increased soil C and N storage in alpine meadows through changes in the species composition and biomass allocation pattern. Although grazing in the warm-season is good for plant diversity conservation and nutrient storage in the topsoil, grazing in the cold season can enhance for C and N storage in deep soil layers (Gao-Lin et al., 2017).

#### 4.2.3 Impacts of grazing intensity on soil organic carbon (SOC) under moist/warm climates

In the MW climatic zone, where both moisture and temperature are high, all GIs have a beneficial impact on SOC. High temperatures increase soil microbial C due to faster decomposition of plant residues and immobilization of products in the microbial biomass. However, Devi et al. (2014) found that only medium GI benefits sub-tropical grasslands by influencing nutrient dynamics and should therefore be prescribed for the management of these grasslands. Da Silva et al. (2014) reported that light GI was a useful management for enhancing C sequestration, whilst high GI led to a reduced number of plant species, plant basal area, and amount of deposited dead plant material. Wright et al. (2004) also reported that long-term grazing at low GI of Bermuda-grass pastures can increase SOC and SON concentrations and could have strong potential for C and N sequestration. This is mainly due to enhanced turnover of plant material and excreta under low GI. Franzluebbers et al., 2000a, Franzluebbers et al., 2000b found that long-term grazed pastures in the Southern Piedmont USA have great potential to restore natural soil fertility, sequester SOC and N and increase soil biological activity compared to other land use management options (e.g. cropping). The processing of forage through cattle and deposition of faeces onto the pasture leads to long-term storage of SOC (Franzluebbers et al., 2000a, Franzluebbers et al., 2000b). In contrast, other studies (e.g. Kieft, 1994, Shrestha and Stahl, 2008) found no consistent impacts of GI on soil C and N, C/N ratios and microbial biomass and respiration rate. There is a lack of quality studies in Middle and West Asia and Africa, and this is a future research requirement.

#### 4.2.4 Impacts of grazing intensity on soil organic carbon (SOC) under dry/cool climates

In the DC climatic zone, where both moisture and temperature are low, low to medium GIs are beneficial for SOC, while the impact of high GI is unknown, since we found no relevant published data. According to Ganjegunte et al. (2005) and Han et al. (2008) low to medium GI is the most sustainable grazing management system to increase SOC in this environment. Han et al. (2008) reported that high GI diminished grass regrowth, decreased litter deposition and decreased SOC. Steffens et al. (2008) reported that sheep grazing at high GI deteriorated physical and chemical parameters of steppe top-soils and depleted SOC and could be improved by reducing GI or excluding from grazing. Further, long-term grazing at different intensity levels significantly reduced SOC and TN in an Inner Mongolian grassland (Li et al., 2008, Ma et al., 2016). Also, soil compaction induced by sheep trampling changes selected soil properties, possibly enhances soil vulnerability to water and nutrient loss, and thereby reduces plant available water, and thus grassland productivity (Zhao et al., 2007). In contrast, Reeder and Schuman (2002) found that grazing at high and low intensities increased SOC, partly due to rapid annual shoot turnover and redistribution of C within the plant-soil system, as a result of changes in plant species composition.

### 4.3 Impacts of grazing intensity on C3/C4 dominated grass or C3-C4 mixed grasslands

Our results show that for C4 dominated grasslands, increased GI, on average, was associated with significantly increased SOC, whilst it significantly decreased SOC for C3 dominated grasslands and C3-C4 mixed grasslands. Similar findings were reported by McSherry and Ritchie (2013). The reason for increased SOC levels under grazed C4-dominated grass, especially in tropical grasslands, is the ability of the grass to adapt and compensate for grazing practices (Ritchie, 2014). C4 grasses adapt to high GI by having many rhizomes and other storage organs that enable them to respond quickly to grass defoliation by animals (McNaughton, 1985, Dubeux et al., 2007). In addition to the warm temperature that encourages macro-decomposers to incorporate plant and animal materials in the soil (Risch et al., 2012), C4-grasses can compensate the loss by sacrificing stems for leaves (Ziter and MacDougall, 2013), and by containing higher levels of lignin and cellulose (Barton et al., 1976). As C4 dominated grasslands would be generally in the moist warm climatic zone, these results are self-consistent.

### 4.4 Impacts of grazing intensity on other selected soil properties (TN, BD and pH)

There were too few data points in each climatic zone to assess the impact of grazing intensity on pH, BD and TN separately for each climatic zone. However, pooling data across all climatic zones suggests that, on average, GI could significantly increase TN and BD but the effect on soil pH was small. Many studies have found higher BD (e.g. Dong et al., 2012, Luan et al., 2014, Abril and Bucher, 1999, He et al., 2011) and high pH (e.g. Su et al., 2005, Pei et al., 2008, Enriquez et al., 2015) in response to high GI in different climatic zones. Grazing intensity increases soil BD and lowers soil moisture content, mainly due to increased animal trampling (He et al., 2011, Zhang et al., 2017), leading to higher denitrification losses (Oenema et al., 1997) and may increase the risk of soil erosion by wind (Kölbl et al., 2011). However, some studies have found lower BD due to GI e.g. Li et al. (2008) and Schuman et al. (1999). High GI was reported to decrease soil pH (Hiernaux et al., 1999, Cui et al., 2005, Zhang et al., 2017). Also, many studies (e.g. Wright et al., 2004, Ganjegunte et al., 2005, Han et al., 2008, Li et al., 2011) have found that GI increases TN, while others suggest it decreases TN (e.g. Li et al., 2008, Ma et al., 2016, Zhou et al., 2017) or results in no change (Schuman et al., 1999).

## 5 Concluding remarks

Overall, the impact of GI on SOC stocks differed between the different climatic zones. Lower GIs increased SOC stocks in three of the four climatic zones (DW, DC and MW), while higher GIs resulted in increased SOC in only one climatic zone (MW). Such climate impacts should be considered in future grassland management and conservation plans. Although our model for predicting biomass production does not take into account extra gains in productivity that can be achieved (promoting increased C sequestration), the benefits (in terms of net GHG emissions) of N use will often be offset by increased losses of non-CO_2_ GHG emissions in the form of N_2_O (particularly at higher GIs). There are also differences between C3, C4 and mixed grasslands in their response to GI, and the TN and BD tend to increase under high GI. Best management practices for GI, therefore, need to be tailored to local bioclimatic conditions to avoid loss of soil carbon. Policy makers in each climatic zone should decide on the level of GI depending on the local climate and pasture types they have. The optimal use of GI and grass species has the potential to significantly increase SOC and SON sequestration, and alters C and N cycling in soil. In addition, the breeding of plants with deeper or more extensive root ecosystems e.g. *Festulolium* (ryegrass x fescue hybrid), which have greater efficiency in resource use, could improve carbon storage, water and nutrient retention, as well as biomass yields (Kell, 2011, Humphreys et al., 2003). Our results have important implications for setting future grassland management policies that account for climate change. Thus, it is essential to consider both climate and grass type (C3/C4) in grazing management decisions to address sustainability of SOC, conservation of biodiversity, reduction of GHG emissions and mitigation of climate change.
